# Identification of key genes as potential diagnostic and therapeutic targets for comorbidity of myasthenia gravis and COVID-19

**DOI:** 10.3389/fneur.2023.1334131

**Published:** 2024-02-07

**Authors:** Liyan Huang, Yao Zuo, Hui Yang, Xiaofang He, Lin Zhang

**Affiliations:** ^1^Department of Neurology, Xuanwu Hospital, Capital Medical University, Beijing, China; ^2^Shandong University, Jinan, Shandong, China; ^3^China Rehabilitation Research Center, Beijing Bo’ai Hospital, Beijing, China; ^4^Department of Neurology, The Second Affiliated Hospital of Guizhou University of Chinese Medicine, Guiyang, China; ^5^Department of Pediatric Intensive Care Unit, Guizhou Provincial People's Hospital, Guiyang, China

**Keywords:** autoimmunity, myasthenia gravis, COVID-19, gene expression profiles, pathological mechanisms

## Abstract

**Introduction:**

Myasthenia gravis (MG) is a chronic autoimmune neuromuscular disorder. Coronavirus disease 2019 (COVID-19) has a significant impact on the health and quality of life of MG patients and may even trigger the onset of MG in some cases. With the worldwide development of the COVID-19 vaccination, several new-onset MG cases and exacerbations following the COVID-19 vaccines have been acknowledged. The potential link between myasthenia gravis (MG) and COVID-19 has prompted the need for further investigation into the underlying molecular mechanism.

**Methods and results:**

The differential expression analysis identified six differentially expressed genes (DEGs) shared by myasthenia gravis (MG) and COVID-19, namely SAMD9, PLEK, GZMB, JUNB, NR4A1, and NR1D1. The relationship between the six common genes and immune cells was investigated in the COVID-19 dataset. The predictive value of the shared genes was assessed and a nomogram was constructed using machine learning algorithms. The regulatory miRNAs, transcription factors and small molecular drugs were predicted, and the molecular docking was carried out by AutoDock.

**Discussion:**

We have identified six common DEGs of MG and COVID-19 and explored their immunological effects and regulatory mechanisms. The result may provide new insights for further mechanism research.

## Introduction

Myasthenia gravis (MG) is a rare autoimmune neuromuscular disorder characterized by fatigable weakness. The incidence of MG is approximately 30 cases per million individuals annually (1). The condition arises due to the presence of antibodies targeting the nicotinic acetylcholine receptors (AChR) or other components at the neuromuscular junction (NMJ), such as muscle-specific tyrosine kinase (MuSK), low-density lipoprotein receptor-related protein 4 (LRP4), and ryanodine receptor (RyR), which consequently leads to the destruction and dysfunction of the NMJ.

Most early-onset myasthenia gravis (EOMG) patients exhibit thymic hyperplasia with ectopic germinal centers. The hyperplastic thymus of MG has been widely recognized as the central location for pathological alterations in MG. Within the inflamed thymus, germinal centers serve as sites where B cells undergo somatic hypermutation, class switching, and subsequent differentiation into plasma cells that produce AChR antibodies. These processes were facilitated by autoreactive T cells. Furthermore, the removal of the inflammatory thymus usually leads to an improvement in the progression of the disease (2).

Viral infection, such as Epstein–Barr virus (EBV), human immunodeficiency virus (HIV), poliomyelitis virus, human T lymphocyte oncovirus, viral pharyngitis, hepatitis C and B viruses, herpes simplex virus, West Nile virus, varicella, and Zika virus, was one of the possible triggers of MG due to molecular mimicry between AChR and viral antigen (3–7). Antibodies against the virus in inflammation may cross-react with the AChR and thus break self-tolerance. While there is yet to be a consensus regarding the causes of MG, the risk factors for MG exacerbation have been well identified, including viral or bacterial infections. When affecting respiratory muscles, exacerbation can lead to myasthenic crisis, which is the main cause of death in MG.

Coronavirus disease 2019 (COVID-19) is a highly contagious type of pneumonia that is attributed to the severe acute respiratory syndrome coronavirus 2 (SARS-CoV-2). Although the major manifestations of COVID-19 are respiratory complications, the symptoms affecting the cardiovascular, hematological, and nervous systems have also been recognized (8, 9). The neurological symptoms of COVID-19 usually affect the central nervous system and manifest as encephalitis, encephalomyelitis, multiple sclerosis, and MOGAD (10). COVID-19 can also induce peripheral neuropathies such as Guillain–Barré syndrome (11–13). As a neuromuscular junction disease, MG has been reported to be associated with COVID-19 as well. Especially noteworthy is a case in which MG manifested as the primary symptom of COVID-19 (14–16). Most patients had elevated serum AChR antibody, except for rare cases with anti-MuSK antibody (17, 18). Despite the fact that the evidence for the link between new-onset MG and COVID-19 infection is rare with only 18 reported cases to date, it is important to note that other coronaviruses, such as Middle East respiratory syndrome coronavirus and SARS, which share structural similarities with SARS-CoV-2, have been widely recognized for their association with neuropathies, myopathies, and neuromuscular disorders (19, 20).

In addition to causing new-onset MG, COVID-19 has also been supposed to induce myasthenia exacerbation and myasthenic crisis (21, 22). The occurrence of MG exacerbation in patients with COVID-19 infection ranges from 10 to 15% (23). Reciprocally, MG may affect oropharyngeal muscles and respiratory muscles, thus worsening swallowing and breathing, which predisposes patients to a more severe infection. Several studies have identified MG as an independent risk factor associated with a poorer prognosis in individuals with COVID-19 (24).

With the widespread implementation of vaccination against COVID-19, numerous cases of MG onset or exacerbation due to the COVID-19 vaccines have been documented (25, 26). To date, it has been reported that some vaccines can induce a new onset or exacerbation of MG, including influenza, Bacillus Calmette-Guérin, human papilloma virus (HPV), and hepatitis B vaccines (27–31). Thus far, there have been a total of 27 recorded cases of MG onset subsequent to receiving the COVID-19 vaccination. MG exacerbation after COVID-19 vaccination occurred in approximately 8% of the patients (32).

Given the potential link between MG and COVID-19, which requires further clarification, identifying the shared molecules involved in both diseases can enhance our comprehension of the underlying pathological mechanism governing their association. In the current study, we aimed to identify the common differential expression genes in MG and COVID-19 and further explore the possible function and the corresponding regulatory miRNAs, transcription factors, and small molecule drugs.

## Methods

### Microarray data reprocessing

**Figure 1:**
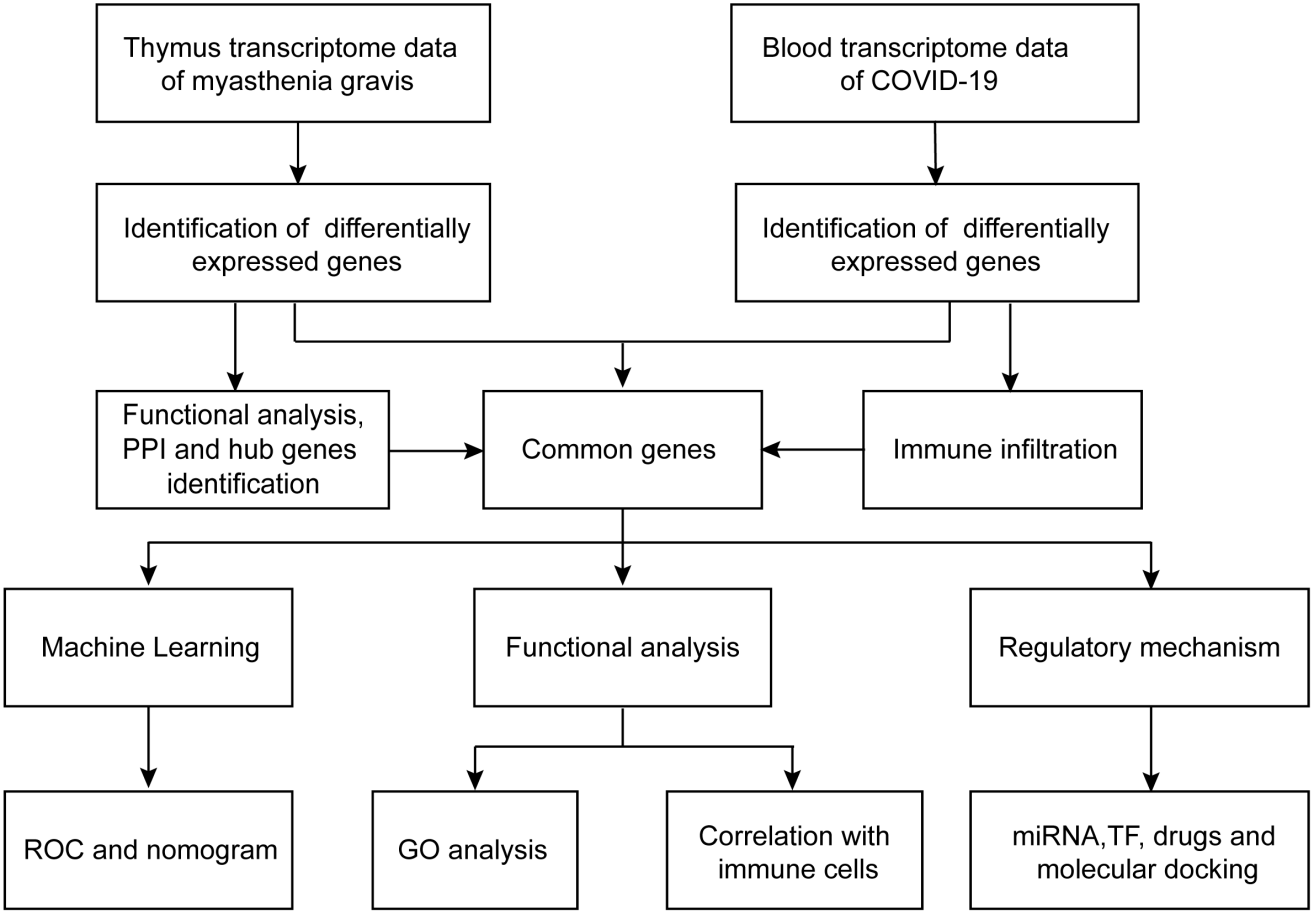
Workflows of the bioinformatic analysis.

**Figure 2:**
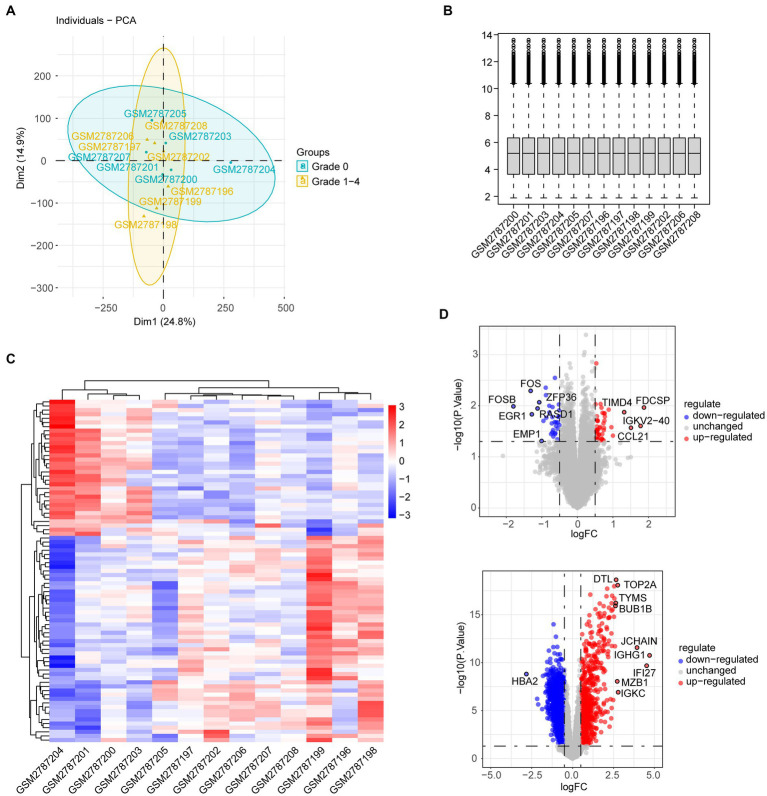
**(A)** PCA of genes from grades 1–4 thymus samples and grade 0 thymus samples of MG patients. **(B)** Boxplot of gene expression in individual MG patients. **(C)** Heatmap shows different gene expression profiles between thymus samples of grades 1–4 and grade 0. **(D)** Volcano plot shows DEGs between thymus samples of grades 1–4 and grade 0.

The workflows are shown in Figure 1. The gene expression profiling datasets GSE103974 of myasthenia gravis and GSE157103 of COVID-19 were obtained from the Gene Expression Omnibus (GEO).^1^ The dataset GSE103974 utilized the GPL17586 Affymetrix Human Transcriptome Array 2.0 platform and GSE157103 utilized the GPL24676 Illumina NovaSeq 6000 platform. GSE103974 contained 7 Grades 1–4 thymus samples from MG patients and 6 Grade 0 thymus samples from MG patients. GSE157103 contained 128 peripheral blood samples from human subjects, consisting of 100 samples from COVID-19 patients and 26 samples from healthy individuals. The selected COVID-19 patients tested positive for the COVID-19 virus and were suffering from moderate to severe respiratory symptoms. The age and sex distribution of the COVID-19 patients is shown in Table 1. The gene expression matrix was annotated using the annotation file downloaded from GEO, and probes lacking gene symbols were excluded. The expression matrix was log2 transformed and normalized using the limma package in R software (version 4.3.0). The boxplot of the data after normalization is shown in Figure 2B. For genes containing multiple probes, the maximum expression level was selected. The difference between groups is displayed by three-dimensional principal component analysis (PCA; Figure 2A).

**Table 1:** The demographic characteristics of patients admitted to intensive care units (ICU) and non-ICU settings with or without COVID-19.

	COVID-19			Non-COVID-19		
	Total	Non-ICU	ICU	Total	Non-ICU	ICU
Case	100	50	50	26	10	16
**Sex *n* (%)**						
Male	62 (62%)	29 (58%)	33 (66%)	13 (50%)	4 (40%)	9 (56%)
Female	38 (38%)	21 (42%)	17 (34%)	13 (50%)	6 (60%)	7 (44%)
**Age**						
Mean (IQR)	61.1 (50.5–74)	59.6 (49–77)	62.6 (55–72.8)	63.8 (53.2–75)	60.4 (50.2–69)	66 (59.8–76.8)

### Differential expression analysis

Differential expression analysis between groups in each dataset was performed using the limma package. Differentially expressed genes (DEGs) were defined with cutoff criteria of a *p* < 0.05 and |Log2 fold-change|(logFC) > 0.5. The heatmap and volcano plot of DEGs were created using the pheatmap and ggplot2 packages.

### Functional enrichment analysis

To obtain the biological functions and signaling pathways of DEGs, a functional enrichment analysis was conducted using Gene Ontology (GO) and the Kyoto Encyclopedia of Genes and Genomes (KEGG). DEGs were uploaded to the online tool Metascape v3.5.20230501.^2^ Terms with a value of *p* < 0.05, a min overlap of ≥3, and an enrichment of ≥1.5 were considered statistically significant. The three categories of GO, namely, biological process (BP), cellular component (CC), and molecular function (MF), were depicted separately. In addition, the ClueGO plugin of Cytoscape was utilized to construct the function enrichment network of DEGs.

### Gene set enrichment analysis

Gene set enrichment analysis (GSEA) is a computational approach employed to assess whether a specified set of genes demonstrates different types of enrichment in two distinct biological states. A value of p below the threshold of 0.05 was considered statistically significant. The R packages “clusterProfiler” and “org.Hs.eg.db” were employed to conduct GSEA of genes.

### DO analysis

To identify the relevant diseases of DEGs, DO analysis was conducted by the clusterProfiler package. A value of p cutoff was set at 0.05.

### Protein–protein interaction network analysis

The protein–protein interaction (PPI) network was constructed using STRING.^3^ Co-expression networks, automated text mining, high-throughput tests, and computational predictions are the main sources of interactions involving both functional and physical relationships. A threshold value of >0.4 for the interaction score was established. The PPI network was designed and visualized using the software Cytoscape v3.10.0. The top 10 genes with the highest degrees were determined using the CytoHubba plugin.

### Machine learning models

The random forest and least absolute shrinkage and selection operator (LASSO) regression algorithms were employed to conduct the additional screening of predictive genes from the pool of six common genes using the random forest and glmnet packages. To detect the diagnostic performance, the receiver operating characteristic (ROC) curves for combined genes were depicted using the pROC package.

### Nomogram construction and validation

The nomogram was developed to assess the incidence of COVID-19 using the rms R package. Each gene contributes to a score. By adding up the individual scores given to the predictors, the cumulative points were obtained for risk assessment. The calibration curve, decision curve analysis (DCA), and clinical impact curve (CIC) were utilized to assess the predictive accuracy of the nomogram.

### Gene regulatory networks

To clarify the associations between common genes and microRNAs (miRNAs), the gene regulatory network was constructed. miRNAs targeting shared genes were predicted by the miRWalk, miRDB, and Targetscan databases. In miRWalk, the filter condition is based on a binding probability value of above 0.95 and a binding site position of 3’UTR. The results of the three databases were intersected and depicted by Cytoscape.

The ChIP-X Enrichment Analysis 3 (ChEA3) database^5^ was used to predict TFs targeting shared genes. The top 10 TFs were identified by average score, and the gene regulatory network was constructed using Cytoscape.

### Drug prediction and molecular docking

The drug prediction was performed using the DsigDB database on the Enrichr web portal.^6^ The 3D structures of small-molecule drugs were obtained from the PubChem database.^7^ The protein crystal structures of targets were obtained from the UniProt database.^8^ Molecular docking was conducted between drug candidates and protein targets using AutoDock software V4.2.6. Docking results were visualized with PyMOL V2.6.0.

### Immune cell infiltration

The gene expression data from the GSE157103 dataset were formatted according to the established guidelines of CIBERSORTx and subsequently uploaded to CIBERSORTx.^9^ We utilized the LM22 gene signature file obtained from CIBERSORTx to examine the blood samples from COVID-19 patients and healthy individuals. This gene signature file allows for the identification of 22 unique subtypes of immune cells. Only samples with a value of *p* < 0.05 were considered to possess precise estimations of immune cell fractions. To further understand the interplay among various types of immune cell infiltration and the relationship between shared genes and immune cells, the Pearson’s correlation analysis was conducted using the corrplot R package.

## Results

### Identification of differentially expressed genes

The gene expression dataset GSE103974 was downloaded from the GEO database. Compared with samples from the Grade 0 group, a total of 83 DEGs were identified in samples from the Grades 1–4 group, with 50 DEGs upregulated and 33 DEGs downregulated. The heatmap visualized the expression pattern of DEGs as well as the level of consistency within the respective groups (Figure 2C). The expression of DEGs is also shown in the volcano plot labeled with the top 10 genes with the highest |logFC| (Figure 2D, upper). Similarly, the volcano plot of DEGs in GSE157103 of COVID-19 is depicted in Figure 2D.

### Functional and pathway enrichment analysis of DEGs

**Figure 3:**
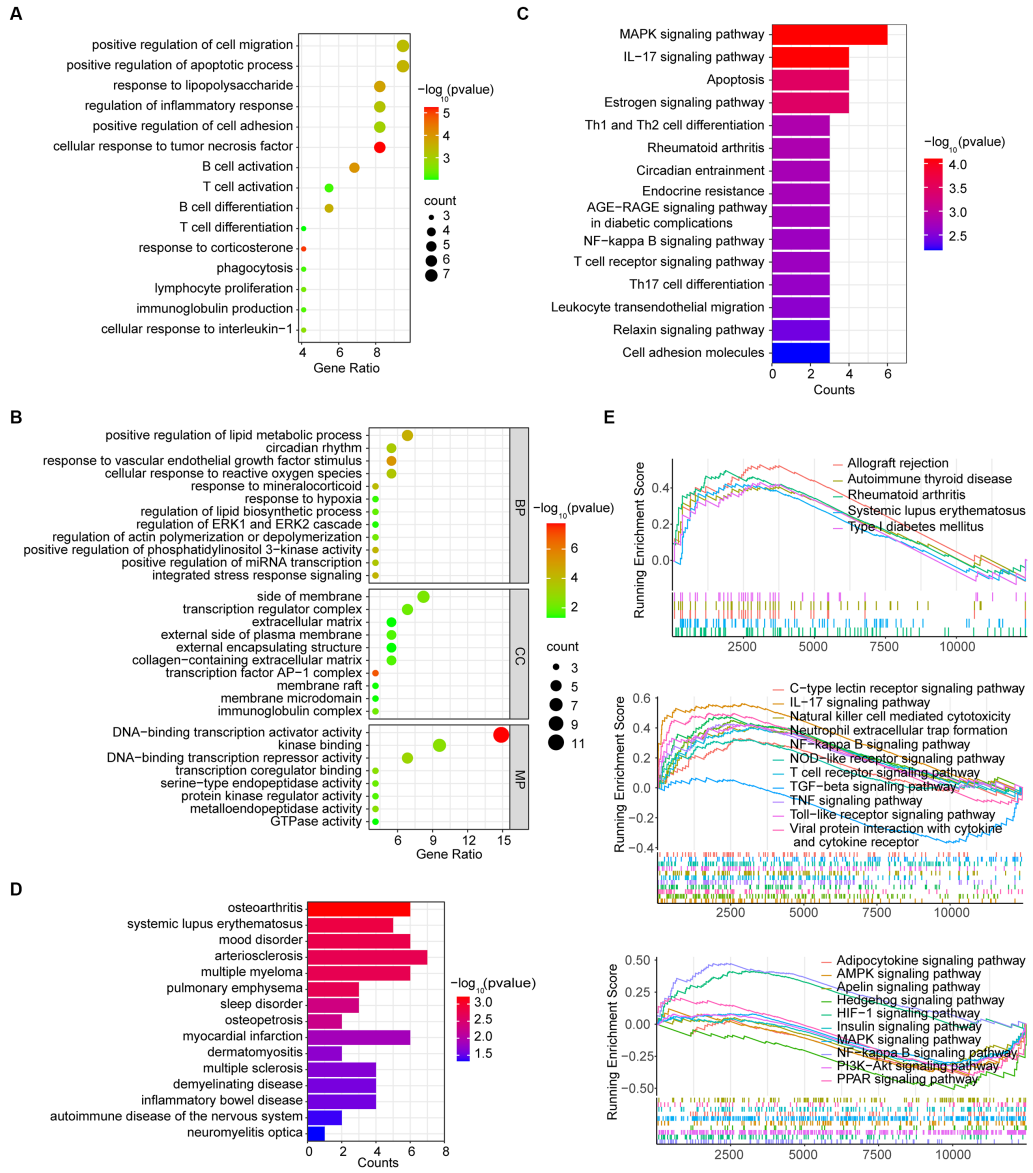
Function analysis of DEGs in the MG dataset. **(A)** Inflammation-associated BP terms by GO analysis of DEGs. **(B)** GO analysis in the BP, CC, and MP categories. **(C)** KEGG analysis of DEGs. **(D)** DO analysis of DEGs. **(E)** GSEA analysis of total genes from grades 1–4 thymus samples and grade 0 thymus samples.

To explore potential biological mechanisms, the enrichment analysis of DEGs in MG was conducted using Metascape. The significant GO function terms for BP associated with inflammation are shown in Figure 3A. Other terms of BP along with terms of CC and MF are illustrated in Figure 3B. The result showed that the hyperplastic thymus exhibited evidence of inflammation with T cell and B cell proliferation, differentiation and activation, immunoglobulin production, cellular response to interleukin-1 and TNF-α, positive regulation of cell adhesion and migration, positive regulation of the apoptotic process, and phagocytosis. Other GO-BP terms included cellular response to ROS, hypoxia, VEGF stimulus, and mineralocorticoid, regulation of ERK1/2 cascade and PI3K activity, integrated stress response signaling, positive regulation of miRNA transcription, positive regulation of lipid metabolic processes and lipid biosynthetic processes, regulation of actin polymerization or depolymerization, and the circadian rhythm. The pathways enriched by GO-MF were related to DNA-binding transcription activator activity, transcription coregulator binding, DNA-binding transcription repressor activity, kinase binding, protein kinase regulator activity, GTPase activity, and serine-type endopeptidase activity. GO-CC analysis indicated that the proteins of DEGs were primarily enriched in the side of the membrane, transcription regulator complex, extracellular matrix, external side of the plasma membrane, external encapsulating structure, collagen-containing extracellular matrix, transcription factor AP-1 complex, membrane raft, membrane microdomain, and immunoglobulin complex. The KEGG analysis showed that DEGs were associated with Th1, Th2, and Th17 cell differentiation, IL-17 signaling pathway, T cell receptor signaling pathway, leukocyte transendothelial migration, cell adhesion molecules, circadian entrainment, endocrine resistance, AGE-RAGE signaling pathway in diabetic complications, NF-κB signaling pathway, relaxin signaling pathway, MAPK signaling pathway, estrogen signaling pathway, and apoptosis, which might resemble the pathological process of rheumatoid arthritis (Figure 3C). The DO analysis of the DEGs indicated that MG is associated with multiple autoimmune disorders and inflammatory diseases (Figure 3D).

### GSEA analysis

We further performed the GSEA analysis of DEGs to find mildly changed biological processes in the MG hyperplastic thymus. The result showed that the pathological process might resemble mildly changed biological processes in other autoimmune diseases such as autoimmune thyroid disease, rheumatoid arthritis (RA), systemic lupus erythematosus (SLE), type 1 diabetes mellitus and allograft rejection (Figure 3E upper). The immunologic process included upregulated natural killer cell-mediated cytotoxicity, neutrophil extracellular trap formation, T cell receptor signaling pathway, viral protein interaction with cytokines and cytokine receptors, IL-17 signaling pathway, TNF signaling pathway, Toll-like receptor signaling pathway, NOD-like receptor signaling pathway, C-type lection receptor signaling pathway, NF-κB signaling pathway, and downregulated TGF-β signaling pathway (Figure 3E middle). Other cellular pathways included the upregulated HIF-1 signaling pathway, downregulated AMPK signaling pathway, MAPK signaling pathway, PI3K-Ark signaling pathway, PPAR signaling pathway, adipocytokine signaling pathway, insulin signaling pathway, apelin signaling pathway, and hedgehog signaling pathway (Figure 3E lower).

### Protein–protein interaction network analysis

**Figure 4:**
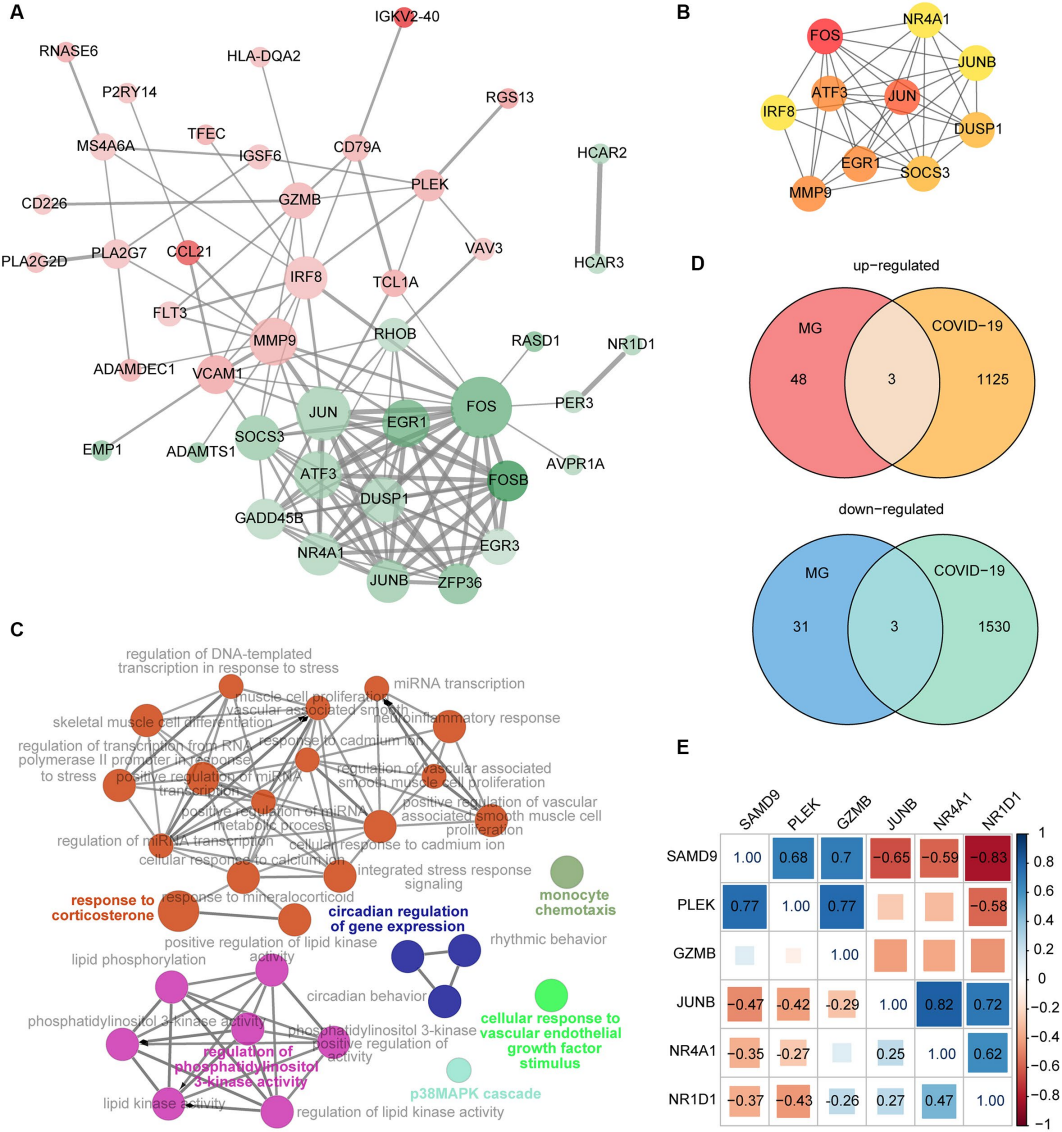
**(A)** The PPI network of DEGs from the MG dataset. The red and green nodes indicate upregulated and downregulated genes, respectively. Color gradients represent the value of |logFC|, and the size of nodes represented the calculated degrees. The width of the edges increases with combined scores. **(B)** The top 10 genes with the greatest degrees were identified by the CytoHubba plugin. The importance of these genes grows as the nodes get redder. **(C)** The function analysis of DEGs from the MG dataset by ClueGO. **(D)** The intersection of DEGs from the MG and COVID-19 datasets. **(E)** Correlation of the six shared genes. The significant relation is labeled with a correlation coefficient.

To further explore the interaction among DEGs, the PPI network was constructed by the STRING database and visualized by Cytoscape. The network has 43 nodes and 123 edges (Figure 4A). The top 10 genes with the highest degrees were identified by CytoHubba, which suggests their potential significance in the progression of MG (Figure 4B). The functional enrichment network generated by ClueGO is illustrated in Figure 4C.

### Identification of common genes

The intersection of DEGs from the MG and COVID-19 datasets is shown in Figure 4D. The shared upregulated genes included SAMD9, PLEK, and GZMB. The shared downregulated genes were JUNB, NR4A1, and NR1D1. These six genes were selected as key genes that may play an important role both in MG and COVID-19. The correlation of the six common genes is displayed in Figure 4E. The significant pairs were labeled with a correlation coefficient.

**Figure 5:**
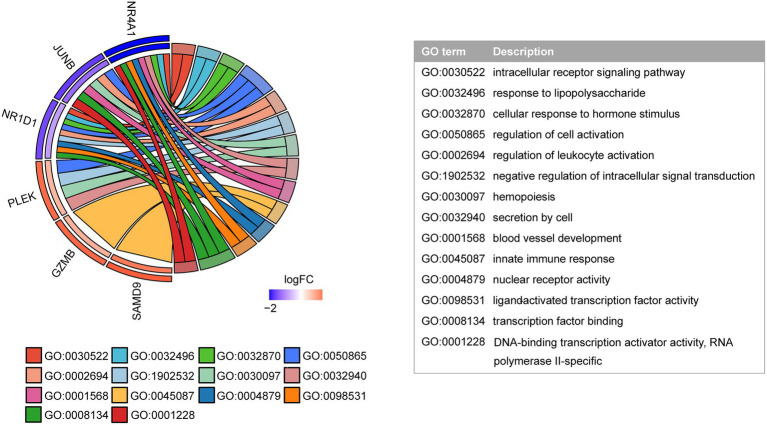
GO function analysis of shared genes. The inner semicircle on the left indicates the logFC of shared genes in the COVID-19 dataset. The outer semicircle indicates the logFC in the MG dataset.

Next, the GO function analysis of the common genes was conducted. As shown in Figure 5, NR4A1 and NR1D1 were both ligand-activated transcription factors involved in response to LPS and cellular response to hormone stimulus. JUNB and NR1D1 were associated with the regulation of leukocyte activation. The genes JUNB and PLEK were implicated in hemopoiesis. NR4A1 and PLEK were linked to secretion by cells. GZMB and SAMD9 were involved in the innate immune response.

### Immunological infiltration in COVID-19

**Figure 6:**
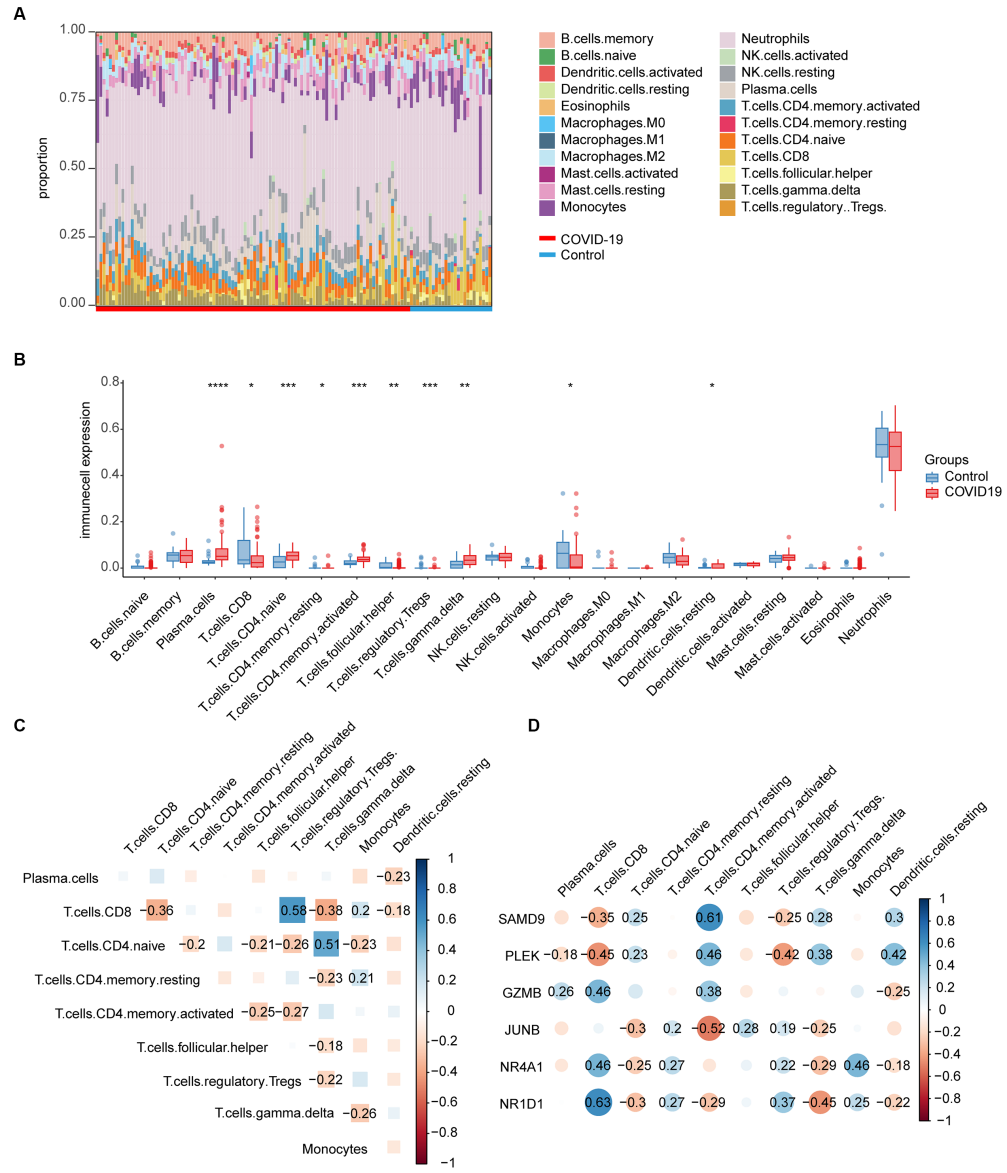
Immunological infiltration in the COVID-19 dataset and its relationship with common genes. **(A)** Immune infiltration distribution of 22 immune cells in 126 samples of GSE157103. **(B)** Abundance of the immune cells in the COVID-19 group and the control group. **(C)** The correlation between the significant immune cells. **(D)** Correlations between common genes and significant immune cells.

The immune infiltration distribution of 22 immune cells in 126 samples of GSE157103 was characterized based on the CIBERSORT algorithm (Figure 6A). The abundance of plasma cells, CD4+ naïve T cells, CD4+ memory-activated T cells, γδT cells, and resting dendritic cells was significantly higher in the COVID-19 group compared to the control group. The abundance of CD8+ T cells, CD4+ memory resting T cells, follicular helper T cells, regulatory T cells, and monocytes was lower in the COVID-19 group than in the control group (Figure 6B). The correlation between the 22 immune cell types is depicted in Figure 6C, where Tregs and CD8+ T cells exhibited the strongest correlation (*r* = 0.58). Next, the correlations between six common genes and significant immune cells were examined (Figure 6D). The results indicated that the three upregulated genes SAMD9, PLEK, and GZMB were positively correlated with CD4+ memory-activated T cells. In addition, SAMD9 and PLEK exhibited a positive correlation with CD4+ naïve T cells, γδT cells, and resting dendritic cells but showed a negative correlation with CD8+ T cells and Tregs. Moreover, GZMB was positively correlated with CD8+ T cells and plasma cells. PLEK was negatively correlated with plasma cells. The downregulated genes JUNB, NR4A1, and NR1D1 were positively correlated with memory-resting CD4+ T cells and Tregs and negatively correlated with naïve CD4+ T cells and γδT cells. In addition, NR4A1 and NR1D1 were positively correlated with CD8+ T cells and monocytes and negatively correlated with resting dendritic cells. JUNB and NR1D1 exhibited a negative correlation with memory-activated CD4+ T cells.

### Machine learning models

**Figure 7:**
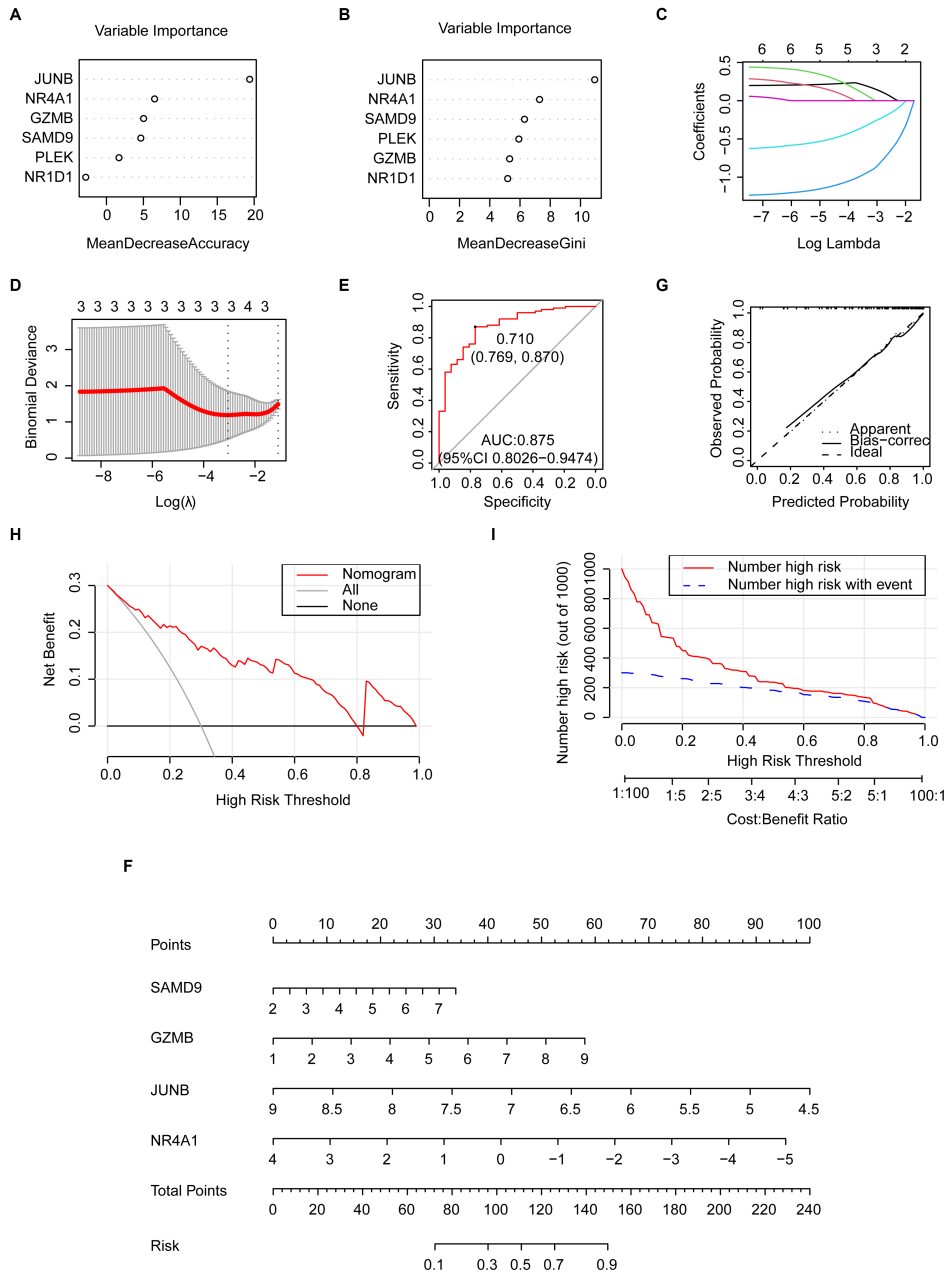
Predictive genes selected by the machine learning model and the predictive nomogram construction. **(A,B)** Importance of common genes in the COVID-19 dataset by random forest. **(C,D)** predictive genes selected by LASSO including SAMD9, GZMB, JUNB, and NR4A1. **(E)** The ROC curve of the four common genes selected by LASSO. **(F)** Nomogram constructed by the selected key genes. **(G)** Calibration curve of the nomogram. **(H)** DCA curve. **(I)** CIC curve.

To mitigate overfitting, the random forest algorithm, least absolute shrinkage, and selection operator (LASSO) regression algorithms were employed. This approach allowed us to identify the most informative genes that possess the most effective diagnostic features among the common genes. The common genes that are considered significant in random forest, as determined by their mean decrease accuracy and mean decrease gini rankings, are visualized in Figures 7A,B. As different log lambdas were taken in LASSO regression, the relative coefficients of shared genes were progressively compressed and approached zero (Figure 7C). Four genes exhibiting non-zero coefficients at the optimal lambda value (lambda.min) were selected, including SAMD9, GZMB, JUNB, and NR4A1 (Figure 7D), which was consistent with the random forest result. The receiver operating characteristic (ROC) curve demonstrated that the combination of the four genes exhibited favorable predictive performance, as indicated by an area under the curve (AUC) value of 0.875 (95% confidence interval [CI]: 0.8026–0.9474). This suggests that the diagnostic model effectively discriminates between COVID-19 patients and control cases (Figure 7E). The specificity and sensitivity were 76.9% and 87%, respectively, at an optimal cutoff value of 0.71.

### Establishment of the predictive nomogram

The nomogram was established to evaluate the risk of COVID-19 occurrence using the four genes from the LASSO model (Figure 7F). The predictive efficiency of the nomogram was evaluated through the analysis of the calibration curve, DCA, and CIC (Figures 7G–I). The calibration curve exhibited a high level of concordance between the predicted and observed values. The DCA curve indicated that the nomogram had high accuracy and could provide evidence for clinical decisions. The CIC curve further indicated that the nomogram could provide a robust clinical benefit for patients.

### The regulatory networks

**Figure 8:**
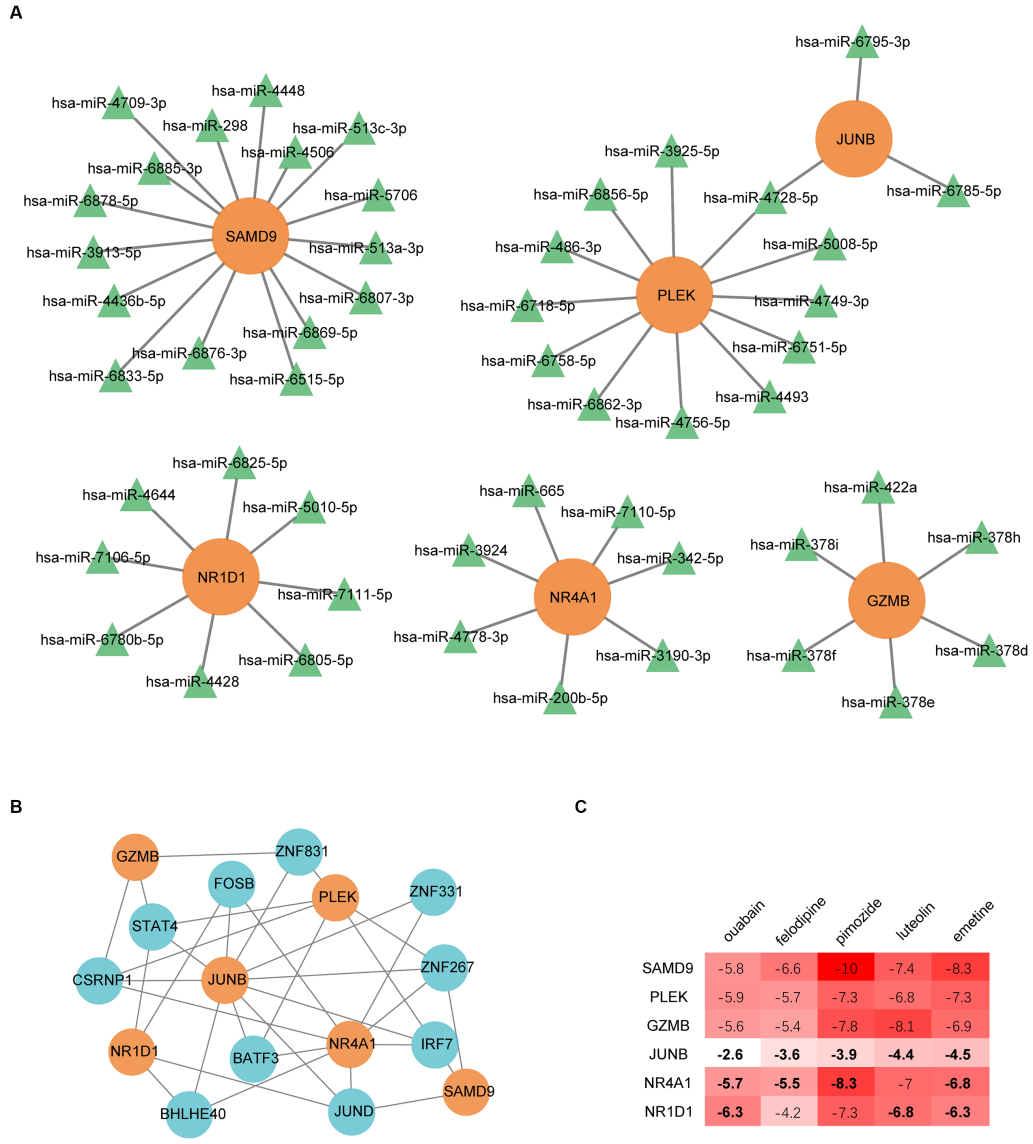
Regulatory networks of common genes. **(A)** miRNA regulatory network. **(B)** The transcription factor regulatory network. **(C)** Binding energy between the proteins expressed by common genes and drug candidates. The bold numbers denote the pairs identified by the DsigDB database on the Enrichr web portal.

We built regulatory networks with miRNA- and TF-genes to learn more about the transcriptional and posttranscriptional mechanisms that control the genes that are shared by MG and COVID-19 patients (Figures 8A,B). A total of 51 miRNAs were obtained by intersecting the predicting results of the miRDB, miRWalk, and TargetScan databases. Among them, hsa-miR-4728-5p simultaneously targets PLEK and JUNB. The top 10 gene-TF interactions were retrieved from ChEA3 by average integrated rank and were illustrated by Cytoscape.

### The relevant autoimmune disorders

The diseases associated with the shared genes of MG and COVID-19 were retrieved from the DisGeNET network^10^ and checked manually (Table 2). All six genes were related to autoimmune diseases, and some were involved in influenza or virus diseases such as GZMB, JUNB, and NR4A1. Of note, GZMB was directly associated with MG. The result suggested an important role for these genes in autoimmune disorders and virus-related diseases.

**Table 2:** Autoimmune diseases associated with the common genes.

Gene symbol	Description	Disease
SAMD9	Sterile alpha motif domain containing 9	Rheumatoid arthritis (33)
PLEK	Pleckstrin	Multiple sclerosis (34), Ulcerative colitis (35)
GZMB	Granzyme B	Myasthenia gravis (36), rheumatoid arthritis (37, 38), systemic lupus erythematosus (39, 40), multiple sclerosis (41), inflammatory bowel diseases (42, 43), type 1 diabetes (44), scleroderma (45, 46), Hashimoto’s thyroiditis (47), influenza (48, 49), virus diseases (50, 51)
JUNB	Jun B proto-oncogene	Systemic lupus erythematosus (52, 53), rheumatoid arthritis (54, 55), psoriasis (56, 57), inflammatory bowel diseases (58–60), systemic sclerosis (61), autoimmune diseases (62, 63), influenza A (64, 65), virus diseases (66), pneumonia (67)
NR4A1	Nuclear receptor subfamily 4	Multiple sclerosis (68, 69), inflammatory bowel diseases (70, 71), rheumatoid arthritis (72), autoimmune diseases (73, 74), influenza (75), pneumonia (76), lung diseases (77, 78)
NR1D1	Nuclear receptor subfamily 1	Inflammatory bowel diseases (79, 80), multiple sclerosis (81)

### Drugs prediction

**Figure 9:**
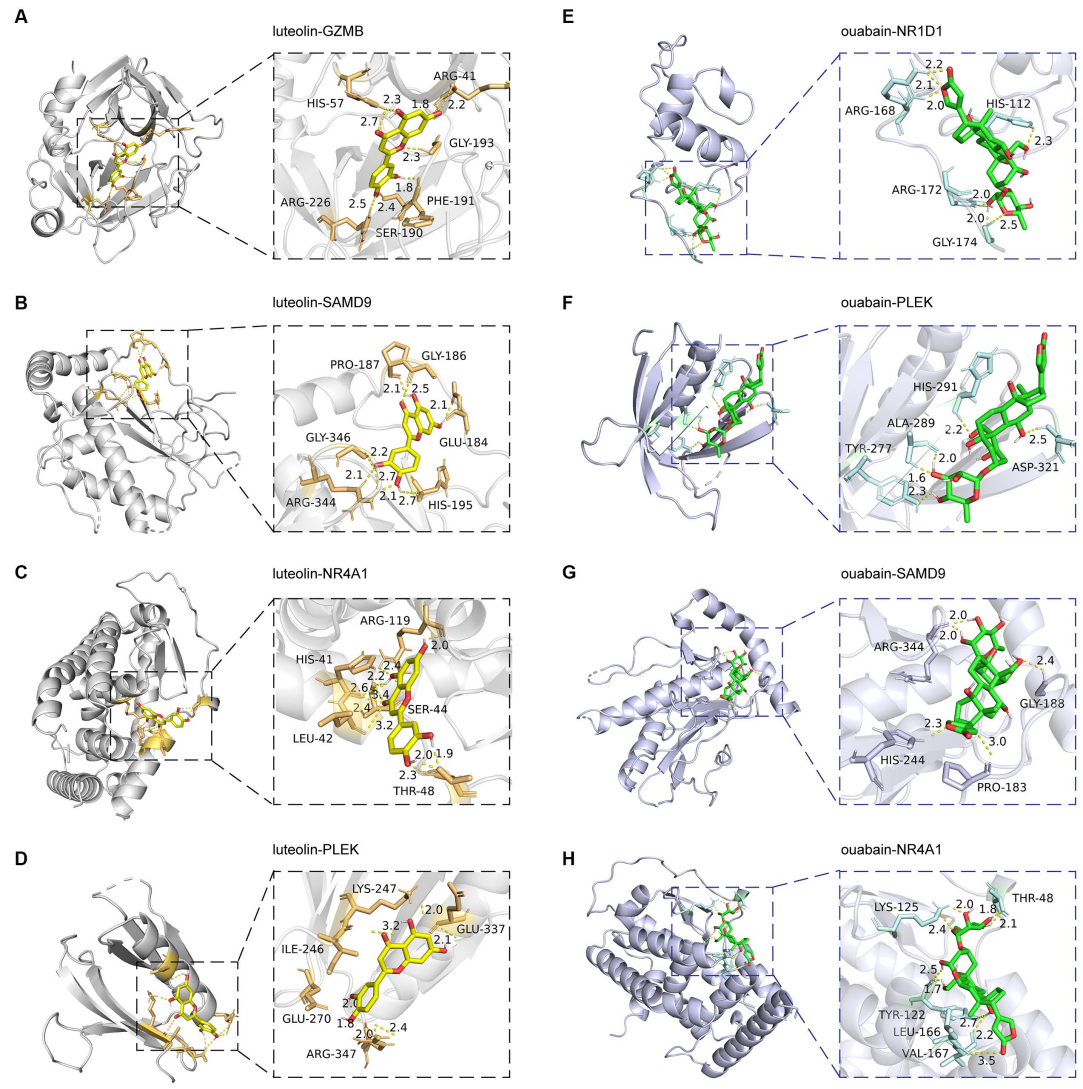
Molecular docking patterns of drugs with proteins expressed by common genes. The left illustrators show luteolin complexed with proteins expressed by **(A)** GZMB, **(B)** SAMD9, **(C)** NR4A1, and **(D)** PLEK. The right illustrators show ouabain complexed with proteins expressed by **(E)** NR1D1, **(F)** PLEK, **(G)** SAMD9, and **(H)** NR4A1.

Small molecular drugs regulating the shared six genes were retrieved from the DsigDB database and ordered by p-value. Ouabain, felodipine, pimozide, luteolin, and emetine were selected from the top 10 drugs through evidence assessment on PubMed (Table 3). AutoDock software was used to forecast the free energy of binding as well as binding modes. The binding energy matrix of the genes and drugs is displayed in Figure 8C. Negative binding energy is indicative of a spontaneous chemical reaction, with the magnitude of the value reflecting the strength of the interaction between the drug and the protein. The binding modes of luteolin and ouabain to the proteins expressed by common genes are depicted in Figure 9.

**Table 3:** List of drug candidates.

Drug	Molecular formula	*p*-value	Adjusted *p*-value	Combined score	Genes
Ouabain	C29H44O12	<0.001	0.017	411.370	NR4A1; NR1D1; and JUNB
Felodipine	C18H19Cl2NO4	<0.001	0.017	959.145	NR4A1 and JUNB
Pimozide	C28H29F2N3O	<0.001	0.017	905.802	NR4A1 and JUNB
Luteolin	C15H10O6	<0.001	0.017	635.699	NR1D1 and JUNB
Emetine	C29H40N2O4	<0.001	0.017	226.613	NR4A1; NR1D1; and JUNB

## Discussion

It has long been acknowledged that viral infection can lead to autoantibody production. The mechanisms of virus-induced autoimmunity include molecular mimicry, epitope spreading, B-cell clonal activation, and bystander activation (82). Some studies have reported the cross-reactivity between AChR and *Escherichia coli*, *Proteus vulgaris*, *Klebsiella pneumonia*, and the herpes simplex virus (83, 84). In addition, viruses can also affect innate immunity by activating Toll-like receptors (TLRs). High levels of TLR3, TRL4, TRL7, and TRL9 have been detected in the thymus of MG patients. TLR3 activation has been proven to upregulate AChR expression and accelerate apoptosis of thymic epithelial cells, which is considered a pivotal upstream event of self-tolerance breakdown in MG. This suggests a strong link between viral infections and MG. SARS-CoV-2 has recently been recognized as a trigger for autoimmune diseases due to molecular mimicry, epitope spreading, immune system overactivation, elevated cytokines, and neutrophil extracellular trap (NET) formation. As for molecular mimicry, it has been established that the more similar peptides the host mammals possess, the more severe the infection of SARS-CoV-2 is (85). In addition, the decreased number and impaired function of Treg cells, along with the elevated levels of IL-17A in COVID-19, have also been considered crucial pathological features of MG (86, 87).

In the present study, we discovered six common genes shared by MG and COVID-19. Among them, NR4A1 and JUNB were also the genes with the highest degrees in the PPI network of MG, indicating their vital function in MG pathogenesis. The functional enrichment analysis that exhibited the typical features of autoimmunity added to the reliability of the DEGs from MG. The correlation of the six shared genes in the two diseases also favored their expression trend. The common genes were associated with the significant immune cells in COVID-19, which implied their crucial roles in immune disorders. In addition, the combination of SAMD9, GZMB, JUNB, and NR4A1 presented excellent diagnostic value in COVID-19. This not only provided an optional diagnostic method for patients suffering from COVID-19 and MG but also emphasized the involvement of these genes in the pathogenesis. Further investigation is warranted to elucidate the connection among these common genes in order to identify the key pathways shared by MG and COVID-19. Due to the limited data, the specific effects of these pivotal genes in MG need to be further explored. Another limitation is the drawbacks of microarrays, including relatively low accuracy, precision, and specificity. Additionally, the experimental setup is susceptible to various factors such as hybridization temperature, genetic material purity, and the amplification process, all of which may influence the assessments of gene expression (88).

SAMD9, known as a potent tumor suppressor gene, encodes a cytoplasmic protein modulating both cell proliferation and apoptosis. SAMD9 expression has been found to be elevated in the Peripheral blood mononuclear cells (PBMCs) of RA patients and PHA-activated Jurkat cells. SAMD9 silencing could enhance Jurkat T cell proliferation and upregulate TNF-α, IL-8, and IFN-γ expression, which indicates mutual regulation between SAMD9 and TNF-α, as SAMD9 is a downstream target of TNF-α signaling (33). The published data to date suggest a compensatory increase of SAMD9 in inflammation, which plays an anti-inflammatory role.

The activation of PLEK results in the production of pleckstrin, which is a PKC substrate expressed by all cells of the hemopoietic system (89). Pleckstrin plays a crucial role in various cellular processes, including cytoskeletal reorganization, cell–cell adhesion, migration, and potentially phagocytosis. It can be upregulated under LPS, IL-1β, or IFN-γ stimulation. Meanwhile, it is important for pro-inflammatory TNF-α and IL-1β production and activation pathways (35). To date, pleckstrin has been implicated in a range of autoimmune and inflammatory diseases such as rheumatoid arthritis, ulcerative colitis, atherosclerosis, T2DM, periodontitis, and CVD (90).

Granzyme B, which is a protease encoded by GZMB in cytotoxic lymphocytes (CTL) and NK cells, is implicated in virus infections and many autoimmune disorders including MG. Consistent with our result, another study also found the presence of granzyme B in the thymus in MG patients but not in normal individuals (36). A hypothesis concerning autoimmunity suggests that the proteolytic cleavage by granzyme B can convert a tolerized self-antigen to novel fragments with newly exposed cryptic determinants, thus triggering the autoimmune response. Diseases with presumptive autoantigens cleaved by granzyme B include lupus erythematosus, rheumatoid arthritis, scleroderma, myositis, and Sjögren’s syndrome, of which more than 20 autoantigens have been identified.

JunB belongs to the AP-1 transcription factor family that assumes a pivotal role in the regulation of cell proliferation, differentiation, apoptosis and inflammation. The crucial involvement of JunB in inflammatory skin diseases has been well documented in the literature. The deletion or reduction of JunB in the epithelial cells of mice led to psoriasis, systemic lupus erythematosus like disease, and myeloproliferative disease, which could probably be explained by elevated cytokines G-CSF and lL-6. Consistent with this report, the skin biopsy specimens of SLE patients also displayed reduced JunB levels and increased IL-6 levels (91). A study revealed that JunB could directly bind to the promotors of IL-6 and G-GSM and inhibit their expression.

NR4A1, also known as Nur77, is a nuclear receptor that can trans-repress other transcription factors, such as NF-κB. The knockdown of NR4A1 leads to the expression of multiple pro-inflammatory cytokines, including IL-1β, IL-6, IL-8, IL-12, IL-17, IFN-γ, TNF-α, GM-CSF, and MCP-1. In addition, the deficiency of NR4A1 also elevates the level of ROS. The absence of NR4A1 can shift macrophages to a M1-dominant phenotype and enhance pro-inflammatory Th1/Th17 differentiation, probably by influencing cell proliferation, apoptosis, and metabolism (92). NR4A1 expression has been observed to be altered in various inflammatory experimental models, including inflammatory bowel disease (IBD), multiple sclerosis (MS), and rheumatoid arthritis.

REV-ERBα, encoded by NR1D1, functions as a ligand-regulated transcription factor that exerts a negative regulatory effect on the expression of core clock proteins. REV-ERBα is primarily expressed in Th17 cells compared with other T cell subtypes. Th17 cells deficient in REV-ERBα result in the elevated expression of Th17-specific genes, including Il17a, Il17f, and Il23r. This phenomenon could be elucidated by the rivalry between REV-ERBα and RORγt, the master regulatory transcription factor of Th17 cells, as they compete for binding to regulatory elements within Th17-specific genes. A GWAS study identified NR1D1 as a multiple sclerosis susceptibility gene. Moreover, REV-ERBα deficiency exacerbated disease severity in a mouse model of multiple sclerosis (93). These studies indicate that REV-ERBα plays a pivotal role in regulating autoimmunity mediated by Th17 cells and is a promising therapeutic target.

In addition to the regulatory miRNA and TF, we also predicted small molecule drugs targeting the proteins expressed by common genes. Ouabain is not only a drug used for the treatment of cardiac insufficiency but also an endogenous hormone with recognized immunomodulatory properties. It has an anti-inflammatory effect by suppressing the expression of IL-6 and TNF-α. Ouabain can restrain the elevated glycolysis by reducing the metabolic regulator HIF1α in the inflammatory process (94).

Felodipine is a pharmacological agent classified as a calcium channel blocker known for its notable antioxidant and anti-inflammatory properties. Felodipine can downregulate the pro-inflammatory cytokine IL-18. IL-18 exerts an important influence on inflammation, partly by inducing TNF-α, IL-1α, IL-1β, and IL-6 production (95). A study using a SARS-CoV-2-specific CAR-T-cell model identified several FDA-approved drugs including felodipine as effective in mitigating COVID-19, possibly by modulating the NF-κB pathway (96).

Pimozide is a dopamine D2 antagonist. D3 and D2 receptor activation induces the adhesion of T cells to fibronectin, a key constituent of the extracellular matrix, mediated by β1 integrin. This adhesion is a characteristic feature observed in activated T cells, facilitating their migration across blood vessels and tissue barriers (97). This evidence suggests that pimozide may have an anti-inflammatory function.

Luteolin is a member of the flavonoid family with antiviral and anti-inflammatory capabilities. Luteolin can block the entrance of SARS-CoV-2 into the host cells and inhibit the cytokine storm caused by SARS-CoV-2 (98). It is also effective in the treatment of experimental autoimmune thyroiditis, adjuvant-induced arthritis, the ulcerative colitis model, the psoriasis model, and PBMC from MS patients. Luteolin’s anti-inflammatory effect involves reducing the proportion of Th1/Th2 and Th17/Treg, promoting the polarization of M2 macrophages, blocking NF-κB, JAK–STAT, AP-1, and TLR signaling pathways, inhibiting NLRP3 inflammasome activation, suppressing pro-inflammatory mediators, and decreasing the intracellular levels of reactive oxygen species (ROS).

Emetine is a natural alkaloid with antiviral activity. Emetine has shown a satisfactory inhibitory effect against both DNA and RNA viruses. This compound has been recognized for its potent anti-SARS-CoV-2 activity, which exhibited sub-micromolar EC50 value. Emetine also exerts an immunomodulatory effect by inhibiting NF-κB, which leads to reduced pro-inflammatory cytokines, including TNF-α, IL-1β, and IL-6 (99).

## Conclusion

In the present study, we identified six shared genes (SAMD9, PLEK, GZMB, JUNB, NR4A1, and NR1D1) of MG and COVID-19 patients through bioinformatic analysis. This study may offer innovative perspectives on the pathogenesis and theoretical foundation for the targeted therapy of the two related diseases.

## Data availability statement

Publicly available datasets were analyzed in this study. This data can be found at: GSE103974: https://www.ncbi.nlm.nih.gov/geo/query/acc.cgi?acc=GSE103974 GSE157103: https://www.ncbi.nlm.nih.gov/geo/query/acc.cgi?acc=GSE157103.

## Ethics statement

The requirement of ethical approval was waived by the medical ethics committees of the Second Affiliated Hospital of Guizhou University of Traditional Chinese Medicine for the studies involving humans because all data are from the GEO public database. The studies were conducted in accordance with the local legislation and institutional requirements. Written informed consent for participation in this study was provided by the participants’ legal guardians/next of kin.

## Author contributions

LH: Conceptualization, Data curation, Formal analysis, Writing – original draft. YZ: Formal analysis, Writing – review & editing. HY: Investigation, Writing – review & editing. XH: Writing – review & editing. LZ: Conceptualization, Writing – review & editing.

## Footnotes

^1^ https://www.ncbi.nlm.nih.gov/geo/

^2^ https://metascape.org/

^3^ https://string-db.org/

^5^ https://maayanlab.cloud/chea3/

^6^ https://maayanlab.cloud/Enrichr/

^7^ https://pubchem.ncbi.nlm.nih.gov/

^8^ https://www.uniprot.org/

^9^ http://cibersort.stanford.edu/

^10^ www.disgenet.org/
